# Association between android-to-gynoid lean mass ratio and advanced cardiovascular-kidney-metabolic syndrome, and the cross-sectional association decomposition of estimated glucose disposal rate

**DOI:** 10.1016/j.clinsp.2026.101170

**Published:** 2026-10-15

**Authors:** Ye Zhang, Rui Zhuang, Zheng Zhuo, Chaofeng Niu, Chenxi Xu, Xuetong Wang, Bing Li, Ce Wang, Lijing Zhang, Meng Li

**Affiliations:** a Dongzhimen Hospital, Beijing University of Chinese Medicine, Beijing, China; b Beijing Institute of Petrochemical Engineering, Beijing, China

**Keywords:** NHANES, AGLR, CKM Syndrome, RCS, eGDR

## Abstract

**Background:**

The Android-to-Gynoid Lean Mass (LM) Ratio (AGLR) reflects the distribution of body LM and may influence the risk of advanced Cardiovascular-Kidney-Metabolic (CKM) syndrome. However, the extent to which Insulin Resistance (IR), as indicated by the estimated Glucose Disposal Rate (eGDR), explains the association between AGLR and advanced CKM syndrome remains unclear. This study aims to elucidate the relationship between AGLR and advanced CKM syndrome and to determine the contribution of eGDR to this association.

**Methods:**

7,399 participants from the 2003‒2006 and 2011‒2018 cycles of the National Health and Nutrition Examination Survey (NHANES) were included in this cross-sectional study. The association between AGLR and advanced CKM syndrome was assessed using weighted Logistic Regression (LR) and subgroup analyses. The potential relationship was further examined using Restricted Cubic Splines (RCS). Sensitivity analyses were conducted to evaluate the robustness of the findings. Cross-sectional decomposition analysis was performed to quantify the contribution of eGDR to the observed associations.

**Results:**

Higher AGLR was associated with stage 4 of CKM syndrome (OR = 1.83, 95% CI: 1.34‒2.50, p < 0.001). For stage 3, the overall trend across quartiles was not significant, indicating a less robust association. Reverse causality cannot be excluded. RCS analysis revealed a positive linear relation (p for nonlinear = 0.681). Subgroup analyses suggested that interactions were statistically insignificant across subgroups. Cross-sectional decomposition analysis showed that eGDR statistically accounted for 33.71% of the association. Notably, given shared components and the cross-sectional design, this should not be interpreted as causal mediation.

**Conclusion:**

LM distribution is relevant to CKM syndrome progression. IR, as reflected by eGDR, may represent a key underlying pathological mechanism.

## Highlights

• AGLR was associated with advanced cardiovascular-kidney-metabolic syndrome.
• eGDR statistically explained 33.7%, without implying causal mediation.
• AGLR and eGDR may improve CKM risk assessment and prevention strategies.

## Background

Cardiovascular-Kidney-Metabolic (CKM) syndrome is a systemic disorder involving Metabolic Risk Factors (MRFs), Chronic Kidney Disease (CKD), and the cardiovascular system.^1^ Its stages (0‒4) reflect progressively worsening multiorgan impairment and an increasing risk of adverse cardiovascular events. Stages 3 and 4 indicate advanced CKM syndrome, representing its most severe manifestation. Nearly 90% of American adults have CKM syndrome (stage 1 or higher), with 15% meeting criteria for advanced stages.^2^ This complex condition contributes to a gradual decline in physical function and quality of life, imposes substantial practical and emotional burdens on families, and generates considerable healthcare and public health costs.

Abdominal obesity is a major risk factor for CKM syndrome,^1^ as it releases mediators that promote inflammation and Oxidative Stress (OS), thereby damaging tissues in the arteries, heart, and kidneys.^1^^,^^3^ Ectopic fat distribution and the metabolic activity of adipose tissue also play key roles in CKM syndrome pathogenesis.^4^ Although Body Mass Index (BMI) is widely used to assess general adiposity, it cannot distinguish fat mass from Lean Mass (LM). The Android-to-Gynoid LM Ratio (AGLR) reflects centrally distributed LM, and an elevated AGLR is associated with a higher risk of cause-specific mortality.^5^^,^^6^ Nevertheless, the clinical relevance of AGLR in CKM syndrome remains unclear.

Insulin Resistance (IR) is a central pathogenic mechanism in obesity-related metabolic disorders.^7^ It also impacts atherosclerosis and renal dysfunction, and is an independent adverse cardiovascular outcome risk indicator.^8^ The estimated Glucose Disposal Rate (eGDR), a noninvasive surrogate measure of IR, is associated with metabolic syndrome, cardiovascular outcomes, and mortality through mechanisms involving chronic inflammation, endothelial dysfunction, and glomerular hyperfiltration.^9^^,^^10^ However, whether eGDR contributes to the relationship between AGLR and CKM syndrome has not been established.

This study leveraged large cross-sectional datasets from the National Health and Nutrition Examination Survey (NHANES) to examine the association between central LM distribution, as reflected by AGLR, and advanced CKM syndrome, and to investigate the proportion of this association explained by eGDR. These analyses aim to provide new insights into the diagnosis and prevention of CKM syndrome and to establish a theoretical basis for developing targeted interventions.

## Methods

### Data source and study population

Data were obtained from NHANES, a nationally representative, stratified, multistage probability survey of the health and nutritional status of the U.S. population. The dataset includes demographic, dietary, examination, questionnaire, and laboratory components. The protocol was approved by the Ethics Review Board (ERB) of the National Center for Health Statistics (NCHS) (Protocol Number: Protocol #98-12, Protocol #2005-06, Protocol #2011-17, Protocol #2018-01), and all participants provided informed consent. Because the present study involved a secondary analysis of de-identified, publicly available data, no additional institutional ethics approval was required.

Participants from six NHANES cycles spanning 2003‒2006 and 2011‒2018 were analyzed, including 59,626 individuals with complete body composition data for the android and gynoid regions. 2007‒2010 Data were excluded because DXA-derived body composition measurements required to calculate AGLR were unavailable. The following exclusion criteria were applied: missing CKM diagnosis data (n = 31,724), age < 45-years (n = 11,915), missing eGDR data (n = 8,563), and missing CKM staging data (n = 25) (Fig. 1). This cross-sectional study was reported in accordance with the Strengthening the Reporting of Observational Studies in Epidemiology (STROBE) Statement (shown in STROBE Checklist).

**Fig. 1:**
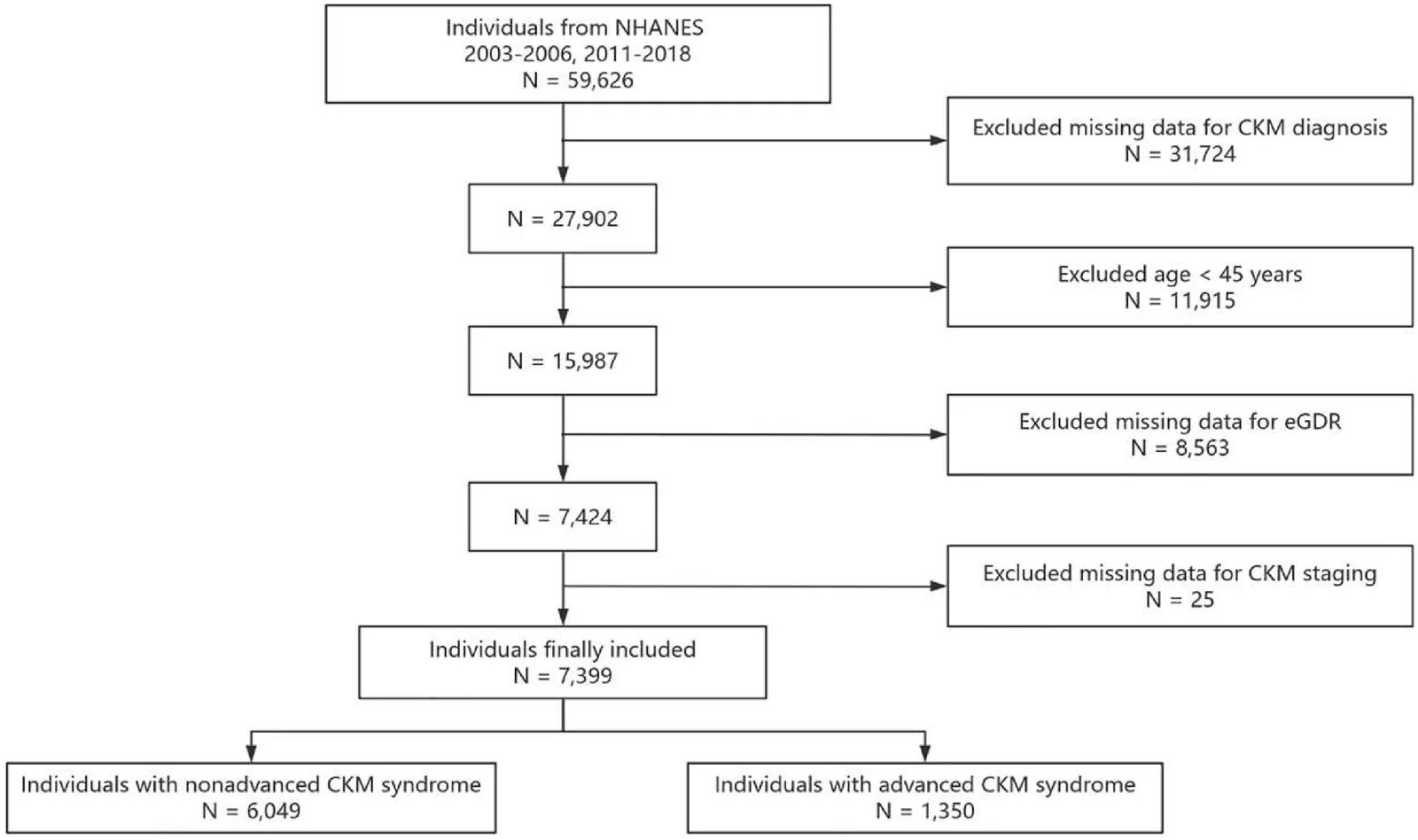
Participant selection flowchart.

### AGLR and eGDR assessment

Body composition was assessed using dual-energy X-Ray Absorptiometry (DXA), with regional definitions based on Hologic APEX.^11^ Specific regions were delineated using anatomical landmarks. AGLR was calculated as android LM divided by gynoid LM.^6^ eGDR, an alternative indicator of IR, was calculated as 21.158−(0.09×waistcircumference[WC,cm])−(3.407×hypertension[yes=1/no=0])−(0.551×HbA1c[%]). Hypertension was defined as: 1) Physician diagnosis, 2) Use of antihypertensive medication, or 3) Systolic Blood Pressure (SBP) ≥140 mmHg and/or Diastolic Blood Pressure (DBP) ≥ 90 mmHg.^12^

### CKM syndrome and staging

CKM syndrome was defined per the American Heart Association (AHA) criteria. Stages 0‒4 were classified as follows:^2^^,^^13^ 0: No CKM syndrome risk factors; 1: Overweight, abdominal obesity, impaired glucose tolerance, or prediabetes, collectively termed excessive or dysfunctional obesity, without other MRFs or CKD; 2: Metabolic disorders (hypertension, metabolic syndrome, hypertriglyceridemia, or diabetes), or moderate-to-high-risk CKD; 3: A forecast 10-year CVD risk ≥20% (computed using the PREVENT equation),^14^ or very high-risk CKD stage (per KDIGO criteria);^15^ 4: Clinical CVDs: coronary heart disease, angina, myocardial infarction, Heart Failure (HF), or stroke.

Staging criteria and PREVENT equations are provided in Tables S1 and S2. Participants were split into advanced syndrome (3‒4) and non-advanced CKM syndrome (0‒2) groups.

### Covariates

Data were obtained from standardized questionnaires, physical examinations, and laboratory analyses of blood and urine samples. Demographic variables included age (continuous, years) and sex (male or female). Socioeconomic variables included Poverty-to-Income Ratio (PIR, ≤ 1.3, 1.3‒3.5, or ≥ 3.5), education (below, equivalent to, or above high school), and marital status (married/living with a partner, never married, or widowed/divorced/separated). Lifestyle factors included smoking and alcohol consumption (yes/no). Health-related covariates included BMI (continuous variable, kg/m^2^), SBP (continuous variable, mmHg); DBP (continuous variable, mmHg); glycated Hemoglobin A1c (HbA1c, continuous variable, %); Serum Creatinine (SCr, continuous variable, mg/dL); estimated Glomerular Filtration Rate (eGFR, continuous variable, mL/min/1.73 m^2^); total fat (continuous variable, g); Total Cholesterol (TC, continuous variable, mg/dL); Triglyceride (TG, continuous variable, mg/dL); High-Density Lipoprotein Cholesterol (HDL-C, continuous variable, mg/dL); Low-Density Lipoprotein Cholesterol (LDL-C, continuous variable, mg/dL), diabetes (yes/no), hypertension, and dyslipidemia. Diabetes was defined as 1) Doctor’s diagnosis, 2) HbA1c ≥6.5%, 3) Fasting glucose ≥ 7.0 mmoL/L, 4) Diabetes medication or insulin taking. Dyslipidemia was defined via: 1) Doctor’s diagnosis, 2) LDL-C ≥130 mg/dL (HDL-C ≤ 40 mg/dL (male), HDL-C ≤ 50 mg/dL (female), TC ≥ 200 mg/dL, TG ≥150 mg/dL, 3) Use of medication to control cholesterol. More details are provided in Table S3.

### Statistical analysis

Considering the intricate sampling, WTSAF2YR was employed. Normality was checked for continuous variables using Kolmogorov-Smirnov tests. For normally distributed ones, results were shown in mean ± Standard Deviation (SD). Differences across cohorts were examined utilizing Student’s *t*-test. For non-normally distributed ones, the median and interquartile range were provided, with differences across cohorts detected employing the Mann-Whitney *U*-test. Categorical variables were in counts and percentages, with statistical evaluations carried out using the Chi-Square test.

The association between AGLR and advanced CKM syndrome was evaluated using weighted Logistic Regression (LR) models, with results reported as Odds Ratios (ORs) and 95% Confidence Intervals (95% CIs). Three models were constructed with stepwise adjustment for potential confounders: Model 1 (crude), Model 2 (adjusted for age and sex), and Model 3 (further adjusted for marital status, education, PIR, smoking, BMI, diabetes, alcohol consumption, and dyslipidemia). Multicollinearity was assessed using Variance Inflation Factors (VIFs), all of which were < 5, indicating no significant multicollinearity (Supplementary Table S4).^16^ The possible dose-response relation was examined through weighted Restricted Cubic Spline (RCS) curves. Subgroup and interaction analyses were conducted across strata of age, sex, PIR, smoking, BMI, diabetes, dyslipidemia, and alcohol consumption. The proportion of the association explained by eGDR was assessed using cross-sectional association decomposition analysis with the association decomposition package in R. A quasi-Bayesian approach with 1,000 Monte Carlo simulations was applied to estimate indirect, direct, and total associations of eGDR. In sensitivity analyses, unweighted LR models were performed to assess the robustness of main findings. The effect of total fat on advanced CKM syndrome was adjusted for, and the association between AGLR and CKM stages 3/4 was evaluated separately to verify the stability of the results. No formal adjustment for multiple comparisons was applied because the analyses were exploratory. All statistical analyses were finished through R 4.5.2. Two-sided p < 0.05 denoted statistical significance.

## Results

### Baseline participant characteristics

7,399 participants were analyzed. Their mean age was 54, and 50% were male. Among these, 1,350 participants (18.25%) were identified with advanced CKM syndrome. Comparative biochemical and clinical characteristics were provided in Table 1. The advanced CKM syndrome patients exhibited a higher proportion of males (58.0%) and a greater proportion aged ≥ 60 (61.0%) than those without the condition. This group also showed higher percentages of being married or living with a partner (56.0%), current smoking (65.0%), and drinking (69.0%). Comorbidities were more prevalent, including diabetes (39.0%), hypertension (81.0%), and dyslipidemia (86.0%). Differences were significant across cohorts by age, marital status, PIR, smoking, blood pressure, gender, HbA1c, SCR, TC, TG, HDL-C, LDL-C, diabetes, drinking, education, hypertension, as well as dyslipidemia.

**Table 1:** Baseline characteristics of participants.

Characteristic	Total (n = 7,399)	Non-advanced CKM (n = 6,049)	Advanced CKM (n = 1,350)	p-value
Age, year	54 (49, 59)	53 (48, 57)	65 (56, 76)	<0.001
Age group, n (%)				<0.001
< 60	5,672 (77%)	5,145 (85%)	527 (39%)	
≥ 60	1,727 (23%)	904 (15%)	823 (61%)	
Gender, n (%)				<0.001
Female	3,734 (50%)	3,161 (52%)	573 (42%)	
Male	3,665 (50%)	2,888 (48%)	777 (58%)	
Marital status, n (%)				<0.001
Married/Living with partner	4,797 (65%)	4,038 (67%)	759 (56%)	
Widowed/Divorced/Separated	1,941 (26%)	1,452 (24%)	489 (36%)	
Never married	661 (8.9%)	559 (9.2%)	102 (7.6%)	
Education, n (%)				<0.001
High school	1,729 (23%)	1,405 (23%)	324 (24%)	
Below high school	1,806 (24%)	1,318 (22%)	488 (36%)	
Beyond high school	3,864 (52%)	3,326 (55%)	538 (40%)	
PIR, n (%)				<0.001
< 1.3	1,972 (27%)	1,489 (25%)	483 (36%)	
≥ 1.3 to < 3.5	2,619 (35%)	2,074 (34%)	545 (40%)	
≥ 3.5	2,808 (38%)	2,486 (41%)	322 (24%)	
Drinking, n (%)				<0.001
No	2,000 (27%)	1,579 (26%)	421(31%)	
Yes	5,399 (73%)	4,470 (74%)	929 (69%)	
Smoking, n (%)				<0.001
No	3,754 (51%)	3,276 (54%)	478 (35%)	
Yes	3,645 (49%)	2,773 (46%)	872 (65%)	
BMI, kg/m^2^	28.2 (24.9, 32.4)	28.2 (24.8, 32.4)	28. 5 (25.3, 32.6)	0.052
BMI group, n (%)				0.043
Normal weight ≤ 25	1,858 (25%)	1,554 (26%)	304 (23%)	
Overweight (25, 30)	2,685 (36%)	2,187 (36%)	498 (37%)	
Obese ≥ 30	2,856 (39%)	2,308 (38%)	548 (41%)	
AGLR	0.52 (0.49, 0.56)	0.52 (0.48, 0.56)	0.55 (0.51, 0.59)	<0.001
AGLR quartiles, n (%)				<0.001
Q1	1,850 (25%)	1,663 (27%)	187 (14%)	
Q2	1,850 (25%)	1,573 (26%)	277 (21%)	
Q3	1,849 (25%)	1,490 (25%)	359 (27%)	
Q4	1,850 (25%)	1,323 (22%)	527(39%)	
eGDR	7.08 (5.22, 9.36)	7.76 (5.53, 9.58)	5.53 (4.32, 6.88)	<0.001
SBP (mmHg)	125 (115, 138)	124 (114, 135)	136 (121, 152)	<0.001
DBP (mmHg)	74 (67, 81)	75 (68, 81)	70 (61, 79)	<0.001
HbA1c (%)	5.60 (5.30, 5.90)	5.60 (5.30, 5.90)	5.70 (5.40, 6.50)	<0.001
SCR (mg/dL)	0.88 (0.73, 1.00)	0.84 (0.71, 1.00)	1.00 (0.80, 1.20)	<0.001
Egfr (mL/min/1.73 m²)	90 (76, 101)	93 (80, 103)	75 (58, 90)	<0.001
Total fat (g)	27,163 (21, 200, 34, 434)	26,984 (21, 024, 34, 330)	27,991 (21, 894, 34, 779)	0.014
TC (mg/dL)	202 (176, 229)	203 (177, 230)	193 (166, 225)	<0.001
TG (mg/dL)	139 (76, 285)	136 (74, 285)	152 (87,287)	0.021
HDL-C (mg/dL)	51 (42, 63)	52 (43, 64)	48 (41, 60)	<0.001
LDL-C (mg/dL)	111 (86, 135)	112 (87, 136)	107 (81, 132)	<0.001
Diabetes, n (%)				<0.001
No	5,957 (81%)	5,130 (85%)	827 (61%)	
Yes	1,442 (19%)	919 (15%)	523 (39%)	
Hypertension, n (%)				<0.001
No	3,617 (49%)	3,366 (56%)	251 (19%)	
Yes	3,782 (51%)	2,683 (44%)	1,099 (81%)	
Dyslipidemia, n (%)				<0.001
No	1,372 (19%)	1,180 (20%)	192 (14%)	
Yes	6,027 (81%)	4,869 (80%)	1,158 (86%)	

CKM, Cardiovascular-Kidney-Metabolic; PIR, Poverty Income Ratio; BMI, Body Mass Index; AGLR, Android-to-Gynoid Lean Mass Ratio; eGDR, Estimated Glucose Disposal Rate; SBP, Systolic Blood Pressure; DBP, Diastolic Blood Pressure; HbA1c, glycated Hemoglobin A1c; SCR, Serum Creatinine; eGFR, estimated Glomerular Filtration Rate; TC, Total Cholesterol; TG, Triglyceride; HDL-C, High-Density Lipoprotein Cholesterol; LDL-C, Low-Density Lipoprotein Cholesterol.

### Association of AGLR with advanced CKM syndrome

Weighted LR analysis demonstrated a significant relation of AGLR (stratified by quartiles) to advanced CKM syndrome (Table 2). In Model 1, Q4 yielded an OR of 4.38 (95% CI: 3.20‒5.82, p < 0.001) for advanced CKM syndrome, with a notable risk rising trend across quartiles (p for trend < 0.001). In Model 3, the relation was statistically significant. Specifically, Q4 had a 70% higher advanced CKM syndrome risk than Q1 (OR = 1.70, 95% CI: 1.21‒2.40, p = 0.003).

**Table 2:** Weighted logistic regression models examining the association between AGLR and advanced CKM syndrome.

	Model 1		Model 2		Model 3	
	**OR (95% CI)**	**p**	**OR (95% CI)**	**p**	**OR (95% CI)**	**p**
**AGLR**						
Q1	Ref	Ref	Ref	Ref	Ref	Ref
Q2	1.70 (1.30, 2.22)	<0.001	1.40 (1.03, 1.90)	0.034	1.14 (0.83, 1.56)	0.422
Q3	2.28 (1.75, 2.99)	<0.001	1.73 (1.28, 2.36)	<0.001	1.18 (0.83, 1.67)	0.357
Q4	4.38 (3.29, 5.82)	<0.001	3.61 (2.65, 4.92)	<0.001	1.70 (1.21, 2.40)	0.003
p for trend	1.62 (1.48, 1,78)	<0.001	1.55 (1.39,1.72)	<0.001	1.97 (1.06,1.34)	0.003

AGLR, Android-to-Gynoid Lean mass Ratio; OR, Odds Ratio; 95% CI, 95% Confidence Interval; BMI, Body Mass Index; PIR, Poverty Income Ratio.

Model 1: Unadjusted. Model 2: Adjusted for gender and age. Model 3: Adjusted further for marital status, education, PIR, smoking, drinking, BMI, diabetes, and dyslipidemia.

RCS analysis, treating AGLR as a continuous variable, indicated a linear dose-response association of AGLR with advanced CKM syndrome (p for nonlinear = 0.681; Fig. 2). These results suggested that the association of AGLR with advanced CKM syndrome was proportional, without evidence of a threshold or nonlinear inflection point. A continuous AGLR rise corresponded to a proportional elevation in the likelihood of the syndrome.

**Fig. 2:**
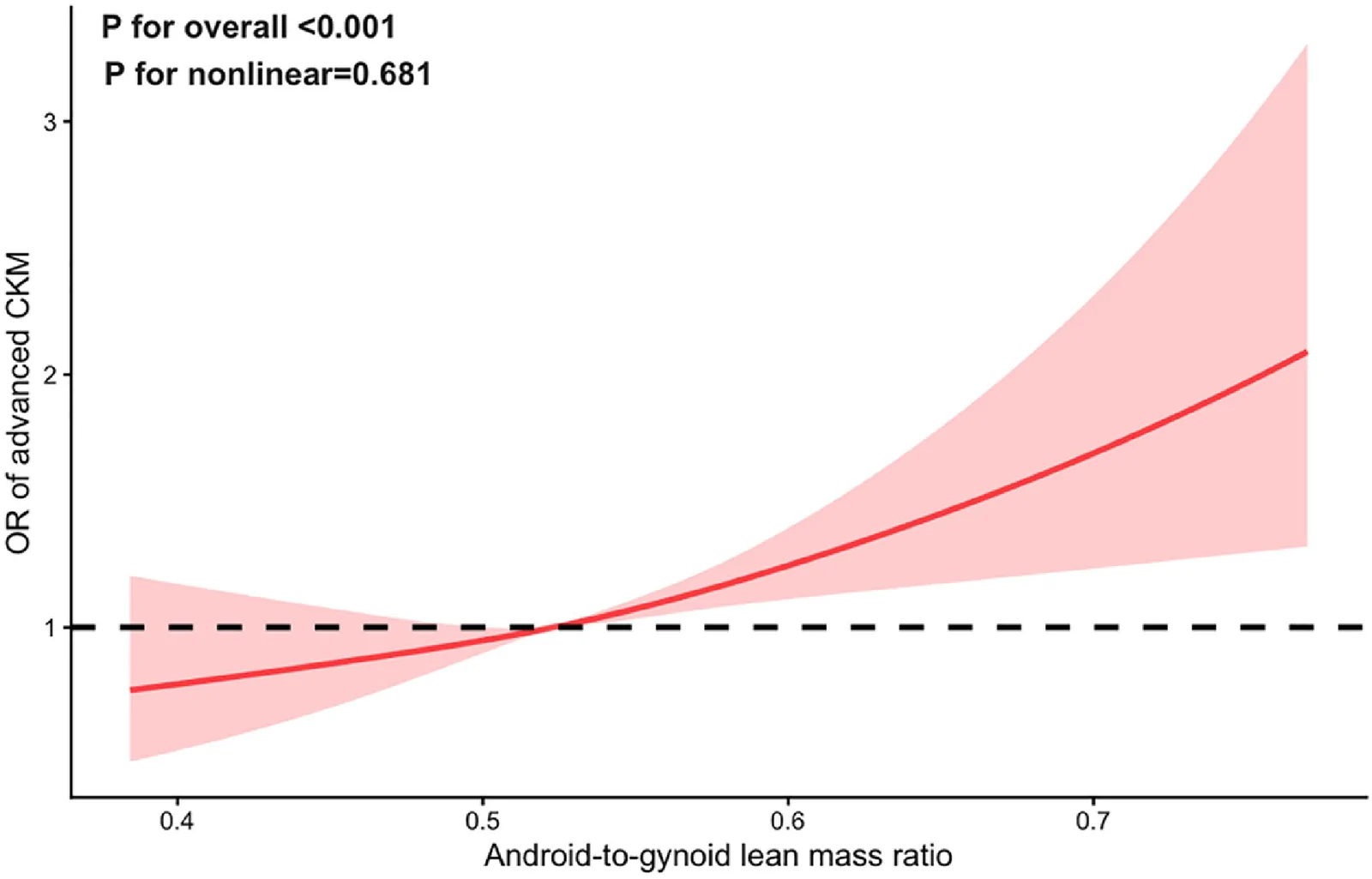
RCS analysis.

### Subgroup analysis and interaction testing

The association of AGLR with advanced CKM syndrome across age, gender, PIR, smoking, drinking, BMI, diabetes, and dyslipidemia was explored in subgroup and interaction analyses (Fig. 3). Interactions were statistically insignificant across subgroups (p for interaction < 0.05 was considered significant), indicating that none of the examined variables significantly modified the association of AGLR with advanced CKM syndrome prevalence. However, the absence of statistically significant interactions may arise from limited power for subgroup analyses rather than true effect homogeneity.

**Fig. 3:**
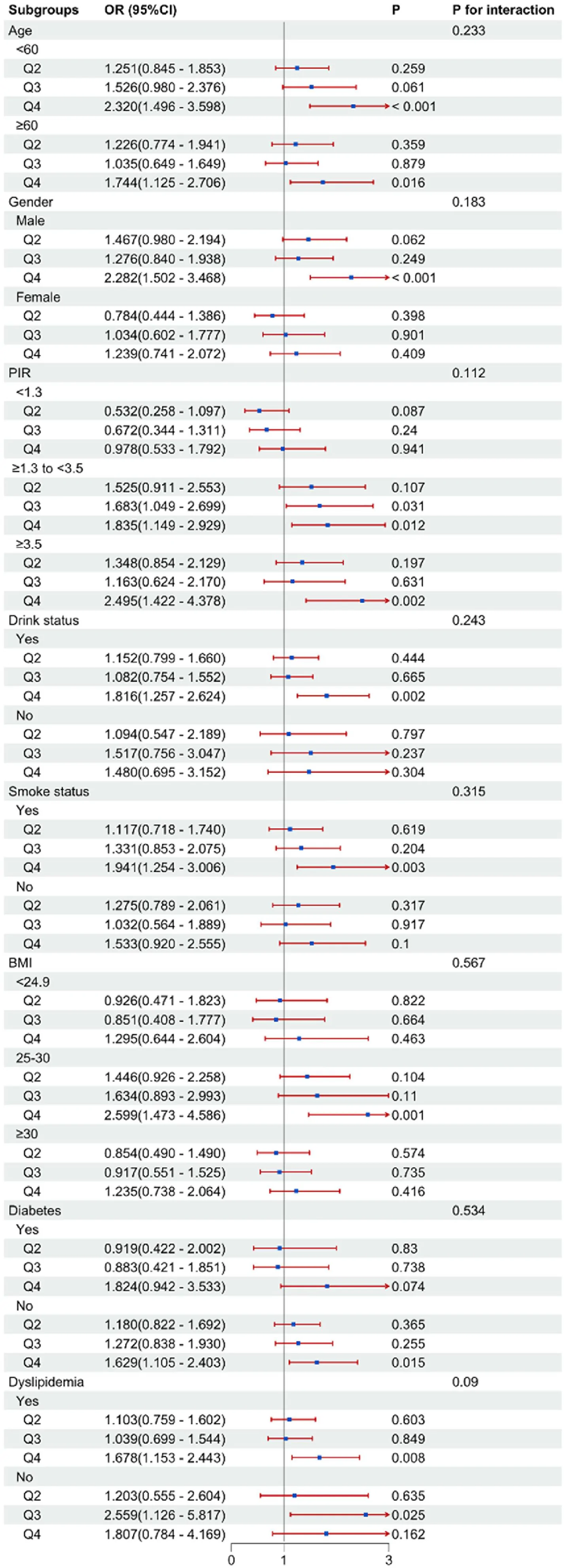
Association of AGLR with advanced CKM syndrome by subgroup. Note: Adjusted for marital status, age, education, PIR, smoking, BMI, diabetes, gender, drinking, and dyslipidemia.

### Proportion between AGLR and CKM explained by eGDR

eGDR partially accounted for the association of AGLR with advanced CKM syndrome (Fig. 4). Results from Model 3 revealed that AGLR had a significant direct association on advanced CKM syndrome. Cross-sectional association decomposition analysis revealed that eGDR accounted for 33.7% of the cross-sectional association of AGLR with advanced CKM. The total association was 0.493 (95% CI: 0.175‒0.526, p < 0.001). The direct association was 0.327 (95% CI: 0.030‒0.361, p = 0.014), and the indirect association was 0.166 (95% CI: 0.115‒0.194, p < 0.001).

**Fig. 4:**
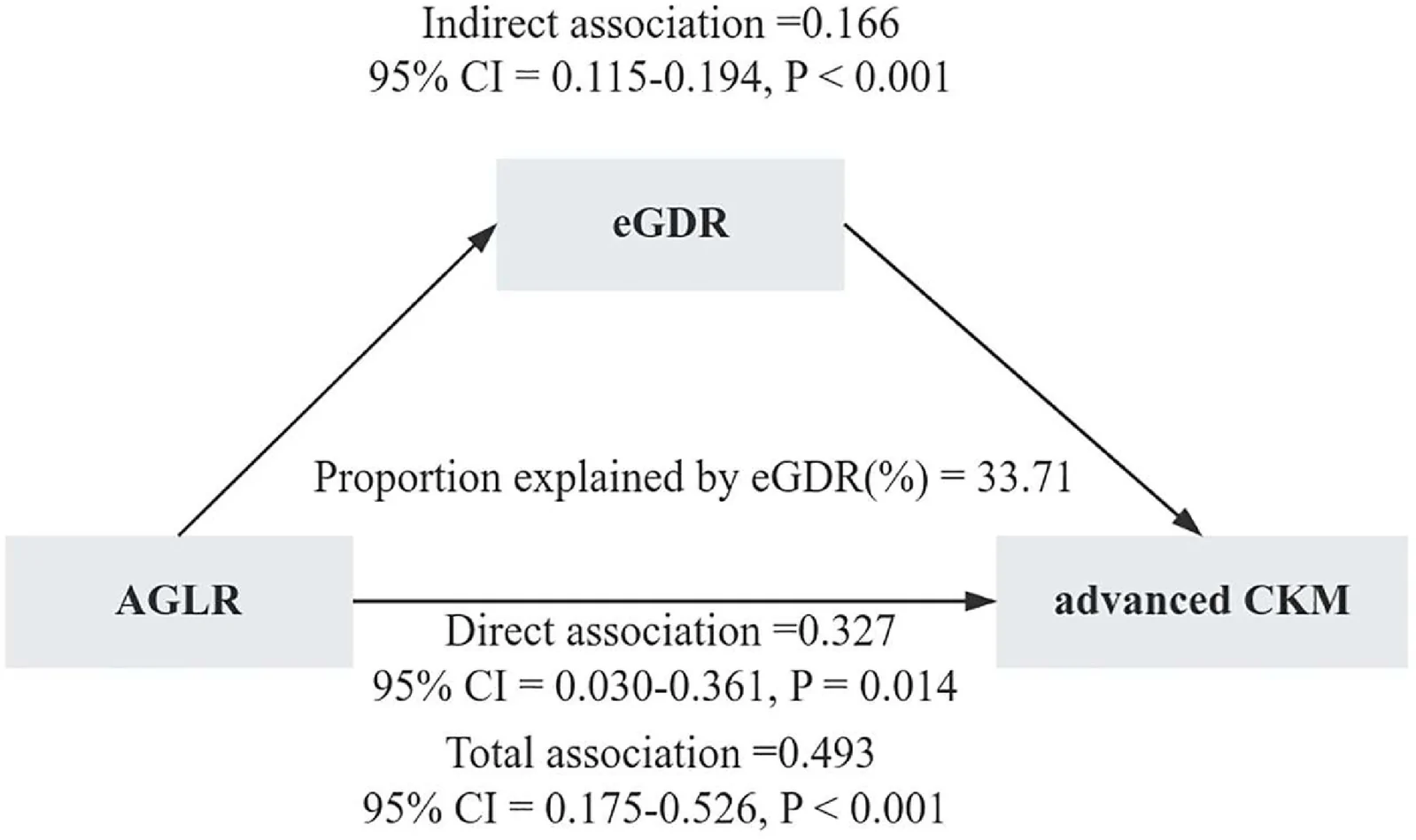
The association decomposition effect of eGDR.

### Sensitivity analysis

To assess the robustness of the association of AGLR with advanced CKM syndrome, non-weighted LR models were applied. The relation remained significant, with higher AGLR quartiles showing elevated advanced CKM syndrome risk relative to lower quartiles (Table 3). This positive relation persisted regardless of covariate adjustment, so the relation is independent of demographic factors, lifestyle behaviors, and comorbidities. Additionally, the separate associations of AGLR with CKM stages 3 and 4 were assessed to verify the robustness of the results. Higher AGLR was associated with stage 4 of CKM syndrome (OR = 1.83, 95% CI: 1.34‒2.50, p < 0.001) (Table 4). For stage 3, the highest quartile showed an OR of 1.80 (95% CI: 1.07‒3.02, p = 0.028), and the p for trend across quartiles was 0.174 (Table 5). After adjusting for total fat in relation to the outcome, the significant association between AGLR and advanced CKM syndrome persisted (Table 6).

**Table 3:** Sensitivity analysis non-weighted logistic regression models examining the association between AGLR and advanced CKM syndrome.

	Model 1		Model 2		Model 3	
	**OR (95% CI)**	**p**	**OR (95% CI)**	**p**	**OR (95% CI)**	**p**
**AGLR**						
Q1	Ref	Ref	Ref	Ref	Ref	Ref
Q2	1.57 (1.29, 1.91)	<0.001	1.23 (0.97, 1.57)	0.087	1.00 (0.77, 1.30)	0.985
Q3	2.14 (1.77, 2.60)	<0.001	1.58 (1.26, 2.00)	<0.001	1.08 (0.84, 1.40)	0.565
Q4	3.54 (2.96, 4.26)	<0.001	3.02 (2.43, 3.77)	<0.001	1.42 (1.10, 1.83)	0.007
p for trend	1.51 (1.43, 1.60)	<0.001	1.46 (1.36, 1.57)	<0.001	1.13 (1.05, 1.23)	0.002

Model 1: Unadjusted. Model 2: Adjusted for gender and age. Model 3: Adjusted further for gender, age, marital status, education, PIR, smoking, drinking, BMI, diabetes, and dyslipidemia.

**Table 4:** Sensitivity analysis weighted logistic regression models examining the association between AGLR and stage 3 of CKM syndrome.

	Model 1		Model 2		Model 3	
	**OR (95% CI)**	**p**	**OR (95% CI)**	**p**	**OR (95% CI)**	**p**
**AGLR**						
Q1	Ref	Ref	Ref	Ref	Ref	Ref
Q2	2.63 (1.56, 4.41)	<0.001	1.52 (0.87, 2.68)	0.143	1.55 (0.89, 2.69)	0.12
Q3	2.64 (1.88, 3.72)	<0.001	1.45 (0.88, 2.38)	0.145	0.84 (0.47, 1.48)	0.536
Q4	6.50 (4.29, 9.84)	<0.001	5.96 (3.82, 9.29)	<0.001	1.80 (1.07, 3.02)	0.028
p for trend	1.75 (1.56, 1.96)	<0.001	1.90 (1.58, 2.27)	<0.001	1.16 (0.94, 1.44)	0.174

Model 1: Unadjusted. Model 2: Adjusted for gender and age. Model 3: Adjusted further for gender, age, marital status, education, PIR, smoking, drinking, BMI, diabetes, and dyslipidemia.

**Table 5:** Sensitivity analysis weighted logistic regression models examining the association between AGLR and stage 4 of CKM syndrome.

	Model 1		Model 2		Model 3	
	**OR (95% CI)**	**p**	**OR (95% CI)**	**p**	**OR (95% CI)**	**p**
**AGLR**						
Q1	Ref	Ref	Ref	Ref	Ref	Ref
Q2	1.76 (1.24, 2.50)	0.002	1.56 (1.10, 2.22)	0.014	1.36 (0.96, 1.93)	0.088
Q3	2.31 (1.68, 3.16)	<0.001	1.86 (1.34, 2.59)	<0.001	1.41 (0.99, 2.01)	0.054
Q4	4.11 (3.04, 5.57)	<0.001	3.24 (2.39, 4.39)	<0.001	1.83 (1.34, 2.50)	<0.001
p for trend	1.57 (1.43,1,73)	<0.001	1.46 (1.33,1.61)	<0.001	1.20 (1.08,1.33)	<0.001

Model 1: Unadjusted. Model 2: Adjusted for gender and age. Model 3: Adjusted further for gender, age, marital status, education, PIR, smoking, drinking, BMI, diabetes, and dyslipidemia.

**Table 6:** Sensitivity analysis adjusted for total fat examining the association between AGLR and advanced CKM syndrome.

	New Model	
	**OR (95% CI)**	**p**
**AGLR**		
Q1	Ref	Ref
Q2	1.12 (0.82, 1.54)	0.475
Q3	1.20 (0.84, 1.73)	0.307
Q4	1.75 (1.25, 2.46)	0.001
p for trend	1.21 (1.08, 1.36)	0.001

New Model: Adjusted for gender, age, marital status, education, PIR, smoking, drinking, BMI, diabetes, dyslipidemia and total fat.

## Discussion

Leveraging NHANES data, this study demonstrates a positive association between AGLR and stage 4 of CKM syndrome. The association with stage 3 was less consistent, as significance was observed only in the highest AGLR quartile and a significant dos-response relationship was not found. Reverse causality cannot be ruled out. Furthermore, eGDR statistically accounted for 33.7% of the association in cross-sectional analysis. However, given shared components and the cross-sectional design, this should not be interpreted as causal mediation.

These findings extend current understanding of the role of LM distribution in CKM syndrome pathogenesis. Reduced muscularity, rather than excess adiposity, has been independently associated with coronary microvascular dysfunction^17^^,^^18^ and is a stronger predictor of diabetes and cardiovascular mortality.^5^^,^^19^^,^^20^ The present study is consistent with these observations and further quantifies the impact of LM distribution on advanced CKM syndrome using AGLR. Previous research has largely focused on adiposity-based indices, such as A Body Shape Index (ABSI) and Body Roundness Index (BRI), which are associated with the onset of diabetes and cardiovascular disease.^21^, ^22^, ^23^ In contrast, AGLR reflects the proportional distribution of LM (fat-free mass, including muscle tissue) in the waist-hip region, directly capturing anatomical LM distribution and thereby complementing and extending prior findings. The results suggest that an elevated AGLR, characterized by greater LM in the abdominal region relative to the hips, may be associated with an increased risk of advanced CKM syndrome. Several mechanisms may explain this relationship. Skeletal muscle functions as an endocrine organ, secreting myokines that regulate energy metabolism, cardiovascular function, and inflammatory responses.^24^ Conversely, Intramuscular ectopic fat accumulation triggers lipotoxicity and IR, promoting local and systemic inflammation and contributing to metabolic dysregulation and CKD development.^25^ Additional evidence indicates that a body composition pattern marked by increased visceral fat and reduced thigh subcutaneous fat exacerbates systemic inflammation, adipokine imbalance, and impaired metabolic homeostasis.^26^ Excess accumulation and dysfunction of ectopic adipose tissue in typically lean tissues represent key etiological drivers of CKM syndrome, promoting IR, systemic inflammation, and OS.^13^^,^^27^ Given the proximity of the android region to visceral fat and abdominal organs, LM in this region may be more susceptible to maladaptive remodeling, thereby intensifying cardiac and renal stress. Consequently, an elevated AGLR may reflect a regionally imbalanced and metabolically unfavorable LM profile that contributes to injury across cardiovascular, renal, and metabolic systems.^28^

Cross-sectional association decomposition analysis showed that eGDR, an index of IR, accounted for 33.71% of the total effect. This finding suggests that the relative distribution of LM may influence CKM syndrome risk partly through its impact on IR. Both the quality and distribution of LM are critical determinants of metabolic, cardiovascular, and renal health.^28^^,^^29^ Lipid accumulation within skeletal muscle exacerbates IR.^24^ Increased android LM, particularly in the context of systemic IR and fat infiltration, may heighten hemodynamic and metabolic stress on central organs.^30^^,^^31^ IR impairs glucose uptake in skeletal muscle, cardiac muscle, adipose tissue, and the kidneys, leading to endothelial dysfunction, nitric oxide inactivation, excessive Reactive Oxygen Species (ROS) production, and progression of cardiovascular disease.^32^ IR also accelerates renal disease progression through mechanisms such as sympathetic activation, sodium retention, and dysregulation of the natriuretic peptide system.^33^ Thus, eGDR may reflect the degree of IR driving CKM syndrome development and progression.

Subgroup analyses revealed no significant effect modification across all subgroups. Nevertheless, previous studies have shown that lifestyle-related factors contribute to the development and progression of CKM syndrome. For instance, components of cigarette smoke, including nicotine and volatile organic compounds, induce endothelial OS and impair vascular function, thereby promoting cardiovascular and renal injury.^34^^,^^35^ Alcohol intake may contribute to CKM syndrome progression using elevated phenylacetylglutamine levels, which are related to increased risk of HF, atherosclerosis, and CKD through inflammatory and endothelial mechanisms.^36^ These findings underscore the potential benefits of lifestyle interventions, such as smoking cessation and reduced alcohol intake, in mitigating CKM syndrome risk.^37^ These insights pave promising avenues for future research aimed at deepening understanding of CKM syndrome.

Our study had several strengths. First, it presents fresh insights into the relation of LM distribution, specifically AGLR, to advanced CKM syndrome risk, while evaluating the proportion of the association explained by eGDR. Second, the large sample size and comprehensive adjustment for multiple confounders enhance the robustness and reliability of the findings. Nevertheless, limitations should be noted. The cross-sectional design precludes causal inference, and the inclusion of only middle-aged and older adults limits generalizability. The association decomposition analysis reflects statistical contributions rather than causal pathways, preventing determination of directionality or confirmation of true mediation; therefore, the findings should be interpreted with caution. Meanwhile, eGDR has not been validated for non-diabetic general populations, which may affect the generalizability of our findings. In addition, several potentially important confounders, including physical activity and specific medication use, were not available, which may have introduced bias. Therefore, our findings should be considered hypothesis-generating rather than definitive, and further prospective studies with comprehensive confounder adjustment shall validate these results. Finally, LM and fat mass represent distinct body components that warrant separate investigation due to their differing effects on health outcomes.^38^, ^39^, ^40^ Owing to data limitations, DXA-derived LM includes non-contractile components and may reflect fat infiltration rather than muscle mass alone. Therefore, the observed associations may not be attributable solely to muscle mass, and reverse causality cannot be excluded, whereby established CVD may contribute to muscle wasting and alterations in body composition. Although total fat was adjusted for in sensitivity analyses to better assess the independent effect of LM distribution, some measurement bias may remain.

## Conclusion

There is a significant positive linear association between AGLR and advanced CKM syndrome, with higher AGLR tertiles associated with increased disease risk. eGDR partially explains this relationship, highlighting IR as a key pathological mechanism linking LM distribution to CKM syndrome. Assessing LM distribution may therefore be important in CKM risk evaluation. Interventions aimed at improving body composition through exercise and nutritional strategies may help reduce AGLR, enhance insulin sensitivity, and slow CKM syndrome progression. In addition, incorporating AGLR and eGDR into population health surveillance may improve CKM syndrome risk monitoring, inform prevention strategies, and support resource allocation for better disease management.

## Data availability statement

All data generated or analyzed during the course of this study came from the NHANES database. The NHANES data utilized in this study were collected with the approval of the NCHS Ethics Review Board (ERB). The NCHS ERB has reviewed and approved the survey protocols for the data cycles used in this analysis.

## Ethics approval

The study was conducted in accordance with the ethical principles outlined in the Declaration of Helsinki. All original NHANES participants provided written informed consent. All analyses were performed in accordance with the NHANES data use agreement of the NCHS. As this study involved the analysis of publicly available, de-identified data obtained from the NHANES database, it did not constitute human subjects research requiring additional approval from IRB. This was an observational cross-sectional study, and clinical trial registration was not applicable. No conflicts of interest were declared.

## Consent to participate

Not applicable.

## Consent to publish

Not applicable.

## Clinic trial number

Not applicable.

## Authors’ contributions

All authors contributed to the study conception and design. Writing-original draft preparation: Ye Zhang; Writing-review and editing: Meng Li; Conceptualization: Rui Zhuang; Methodology: Zheng Zhuo; Formal analysis and investigation: Ye Zhang, Chaofeng Niu, Chenxi Xu; Funding acquisition: Lijing Zhang; Resources: Xuetong Wang, Bing Li, Ce Wang; Supervision: Lijing Zhang, and all authors commented on previous versions of the manuscript.

All authors read and approved the final manuscript.

## Funding

This research was supported by Beijing Natural Science Foundation (L259079).

## Declaration of competing interest

The authors declare no conflicts of interest.
