# Supplementary material for: Association between android-to-gynoid lean mass ratio and advanced cardiovascular-kidney-metabolic syndrome, and the cross-sectional association decomposition of estimated glucose disposal rate
Source: Clinics (Sao Paulo). 2026 Oct 15;81:101170. doi: 10.1016/j.clinsp.2026.101170 (PMC13693339; doi:10.1016/j.clinsp.2026.101170)
Supplement: Supplementary file 1 [file mmc1.docx]

**CLINICS-D-26-00487**

**Supplemental Materials**

**Table S1** Definitions of CKM syndrome stages.

| **CKM Syndrome Stages** | **Definition** |
| --- | --- |
| Stage 0 | Individuals with normal Body Mass Index (BMI) (< 23 kg/m^2^ for individuals with Asian ethnicity and < 25 kg/m^2^ for other racial and ethnic groups), normal waist circumference (< 80 and < 90 cm for women and men with Asian race, respectively, and < 88 and < 102 cm for women and men in all other race and ethnicity categories, respectively) who did not meet criteria for the other stages. |
| Stage 1 | Individuals with an elevated BMI (≥ 23 kg/m^2^ for individuals with Asian race and >25 kg/m^2^ for all other race and ethnic groups), an elevated waist circumference (≥ 80 and ≥ 90 cm for women and men with Asian race, respectively, and ≥88 and ≥ 102 cm for women and men in other race and ethnicity categories, respectively), or prediabetes (defined as a glycated hemoglobin of 5.7% to < 6.5% or a fasting blood glucose of 100 mg/dL to < 126 mg/dL). |
| Stage 2 | Individuals with metabolic risk factors or moderate-to-high-risk CKD according to KDIGO criteria, as recommended by the AHA.[1] Qualifying metabolic risk factors included elevated fasting serum triglycerides (≥ 135 mg/dL), hypertension, diabetes, or metabolic syndrome (≥ 3 of the following: elevated waist circumference, low high density lipoprotein cholesterol level [< 40 mg/dL or < 50 mg/dL for men or women, respectively], fasting serum triglycerides ≥ 150 mg/dL, elevated blood pressure [systolic blood pressure ≥ 130, diastolic blood pressure ≥ 80 mmHg, and/or use of blood pressure-lowering medications], or prediabetes). CKD stages were identified based on eGFR and UACR. |
| Stage 3 | Individuals with very-high-risk KDIGO CKD stages or a high-predicted 10-year CVD risk. 10-year cardiovascular risk was estimated with the AHA Predicting Risk of CVD EVENTs (PREVENT) equations.[2] High risk was defined as ≥ 20% 10-year CVD risk. |
| Stage 4 | Individuals based on self-reported established cardiovascular disease (coronary heart disease, angina, heart attack, heart failure, and stroke). Atrial fibrillation and peripheral artery disease were not included, as these data were not available. |

CKM, Cardiovascular-Kidney-Metabolic; BMI, Body Mass Index; CKD, Chronic Kidney Disease; KDIGO, Kidney Disease Improving Global Outcomes; AHA, American Heart Association; eGFR, Estimated Glomerular Filtration Rate; UACR, Urinary Albumin-to-Creatinine Ratio; CVD, Cardiovascular Disease.

**Table S2** Detailed algorithm of the simplified 10-year CVD risk models.

| **Gender** | **Equation** |
| --- | --- |
| Male | log-Odds = -3.031168 + 0.7688528 × (age – 55) /10 + 0.0736174 × (TC – HDL – 3.5) – 0.0954431 × (HDL –1.3) /0.3 – 0.4347345 × (min(SBP, 110) – 110) /20 + 0.3362658 × (max(SBP, 110) – 130) /20 + 0.7692857 × (if diabetes) + 0.4386871 × (if current smoker) + 0.5378979 × (min(eGFR, 60) – 60) / -15 + 0.0164827 × (max(eGFR, 60) –90) / -15 + 0.288879 × (if using anti-hypertensive medication) – 0.1337349 × (if using statin) – 0.0475924 × (if using antihypertensive medication) × (max(SBP, 110) – 130) /20 + 0.150273 × (if using statin) × (TC – HDL – 3.5) – 0.0517874 × (age – 55) /10 × (TC – HDL – 3.5) + 0.0191169 × (age –55) /10 × (HDL – 1.3) /0.3 – 0.1049477 × (age – 55) /10 × (max(SBP, 110) – 130) /20 – 0.2251948 × (age – 55) /10 × (if diabetes) – 0.0895067 × (age – 55) /10 × (if current smoker) –0.1543702 × (age – 55) /10 × (min(eGFR, 60) – 60) / -15  Risk = exp(log-Odds) / (1 + exp(log-Odds)) |
| Female | log-Odds = -3.307728 + 0.7939329 × (age – 55) /10 + 0.0305239 × (TC – HDL – 3.5) – 0.1606857 × (HDL –1.3) /0.3 – 0.2394003 × (min(SBP, 110) – 110) /20 + 0.360078 × (max(SBP, 110) – 130) /20 + 0.8667604 × (if diabetes) + 0.5360739 × (if current smoker) + 0.6045917 × (min(eGFR, 60) – 60) / -15 + 0.0433769 × (max(eGFR, 60) – 90) / -15 + 0.3151672 × (if using anti-hypertensive medication) –0.1477655 × (if using statin) – 0.0663612 × (if using antihypertensive medication) × (max(SBP, 110) – 130) /20 + 0.1197879 × (if using statin) × (TC – HDL – 3.5) –0.0819715 × (age – 55) /10 × (TC – HDL – 3.5) + 0.0306769 × (age – 55) /10 × (HDL – 1.3) /0.3 – 0.0946348 × (age – 55) /10 × (max(SBP, 110) – 130) /20 – 0.27057 × (age – 55) /10 × (if diabetes) – 0.078715 × (age – 55) /10 × (if current smoker) – 0.1637806 × (age – 55) /10 × (min(eGFR, 60) – 60) / -15  Risk = exp(log-Odds) / (1 + exp(log-Odds)) |

The 10-year CVD risk was calculated from the American Heart Association Predicting Risk of CVD EVENTs (PREVENT) equation.[2]

CVD, Cardiovascular Disease; eGFR, Estimated Glomerular Filtration Rate; HDL, High-Density Lipoprotein; TC, Total Cholesterol; SBP, Systolic Blood Pressure.

**Table S3** Category and definition of the variables.

| **Variables** | **Variable classification** | **Original data** |
| --- | --- | --- |
| Gender | Female, Male | ‒ |
| Age | 20‒39, 40‒59, ≥60 | ‒ |
| Marital status | Married/Living with partner | ‒ |
|  | Widowed/Divorced/Separated | ‒ |
|  | Not married | ‒ |
| Education levels | Less than high school | Less Than 9^th^ Grade, 9‒11^th^ Grade (Includes 12^th^ Grade with no diploma) |
|  | High school | High School Grad/GED or Equivalent |
|  | More than high school | Some College or AA degree, College Graduate or above |
| PIR | 0‒1.29, 1.30‒3.49, ≥3.50 |  |
| Drink Status | No | Less than 12 alcohol drinks 1-year |
|  | Yes | More than 12 alcohol drinks 1-year |
| Smoke Status | No | Less than 100 cigarettes in life |
|  | Yes | More than 100 cigarettes in life |
| BMI | Normal weight | ≦25 kg/m^2^ |
|  | Overweight | 25‒30 kg/m^2^ |
|  | Obese | ≥30 kg/m^2^ |
| Diabetes | No | If both of the following conditions are satisfied, it is YES, and vice versa: 1) Doctor told you have diabetes; 2) Glycohemoglobin HbA1c(%) ≥6.5; 3) Fasting glucose (mmoL/L) ≥7.0; 4) Use of diabetes medication or insulin. |
|  | Yes |  |
| Hypertension | No | If one of the following conditions is met, it is YES, and none of them is met, it is NO: 1) Doctor told you have hypertension; 2) Use of antihypertensive medication; 3) Systolic blood pressure ≥140 mmHg, diastolic blood pressure ≥90 mmHg. |
|  | Yes |  |
| Dyslipidemia | No | If one of the following conditions is met, it is YES, and none of them is met, it is NO: 1) Doctor told you have dyslipidemia; 2) Low-density lipoprotein cholesterol ≥130 mg/dL (high-density lipoprotein cholester ≦40 mg/dL if male, high-density lipoprotein cholesterol ≦50 mg/dL if female), total cholesterol ≥200 mg/dL, triglyceride ≥150 mg/dL; 3) Use of medication to control cholesterol. |
|  | Yes |  |

PIR, Poverty Income Ratio; BMI, Body Mass Index.

**Table S4** Multicollinearity evaluation of variables through Variance Inflation Factor (VIF) analysis.

| **Variable** | **GVIF** | **Df** | **GVIF^1/2Df^** |
| --- | --- | --- | --- |
| Age | 1.213 | 1.213 | 1 |
| Gender | 1.233 | 1.233 | 1 |
| Marital status | 1.141 | 1.141 | 2 |
| Education level | 1.224 | 1.224 | 2 |
| PIR | 1.276 | 1.276 | 2 |
| Drink status | 1.231 | 1.231 | 1 |
| Smoke status | 1.195 | 1.195 | 1 |
| BMI | 1.221 | 1.221 | 1 |
| AGLR | 1.169 | 1.169 | 3 |
| Diabetes | 1.176 | 1.176 | 1 |
| Hypertension | 1.033 | 1.033 | 1 |
| Dyslipidemia | 1.020 | 1.020 | 1 |

PIR, Poverty Income Ratio; BMI, Body Mass Index. AGLR, Android-to-Gynoid Lean mass Ratio.

**References**

1. Kidney Disease: Improving Global Outcomes (KDIGO) CKD Work Group. KDIGO 2024 Clinical Practice Guideline for the Evaluation and Management of Chronic Kidney Disease. Kidney Int. 2024;105(4S):S117-S314.

2. Khan SS, Matsushita K, Sang Y, Ballew SH, Grams ME, Surapaneni A, et al. Development and validation of the American Heart Association’s PREVENT equations. Circulation. 2024;149(6):430-49.
